# Spatial–Temporal Variations of Water Quality in Urban Rivers after Small Sluices Construction: A Case in Typical Regions of the Taihu Lake Basin

**DOI:** 10.3390/ijerph191912453

**Published:** 2022-09-29

**Authors:** Feng Lan, Wang Haisen, Yan Yan

**Affiliations:** 1 College of Civil Engineering, Nanjing Forestry University, Nanjing 210037, China; 2 College of Environment and Biology, Nanjing Forestry University, Nanjing 210037, China; 3 Taihu Water Pollution Prevention and Control Research Center, Jiangsu Provincial Academy of Environmental Science, Nanjing 210042, China

**Keywords:** sluices and dams, urban rivers, water pollution, Taihu Lake, total phosphorus, total nitrogen

## Abstract

Urban river pollution is considered a ‘necessary evil’ consequence of disproportionate developmental expansion in metropolises. Unprecedented expansion and anthropic activities lead to the deterioration of urban rivers with municipal and industrial sewage. The construction of sluices is one of the irrefutable parts of the process. In order to prevent floods and drought, many cities build sluices and dams in rivers to balance water quantity in different seasons. To explore the change characteristics of the water quality in urban rivers after the construction of sluices and dams, the change in the total phosphorus (TP) and total nitrogen (TN) concentrations upstream and downstream of rivers was investigated under the condition of sluices closure in Wuxi. According to the results, when the sluices were closed, the pollutants of TP and TN would accumulate upstream in rivers, which caused the water quality in the upper reaches to be worse than that in the lower reaches. Specifically, the TN and TP concentrations downstream of urban rivers in Wuxi were approximately 14.42% and 13.80% lower than those upstream when the sluices were closed. Additionally, the water quality in urban rivers was usually better in summer and autumn than in the other seasons, showing obvious seasonality after the construction of the sluices. The research will provide a theoretical basis for future sluice operation and the water resources management of urban rivers.

## 1. Introduction

Rivers are an integral part of urban ecosystems [1]. Rapid urbanization and economic development have caused urban river pollution globally, and human activities directly or indirectly lead to changes in river environments. Under natural flow conditions, river water can self-purify, while during the process of urban expansion, the water quality of the river is more or less affected, and the river cannot fully play its original function [2]. In the last decade, the number of hydraulic structures such as sluices and dams has increased with rapid urbanization. It is expected that by 2025, these structures will be present in 70% of rivers around the world [3,4]. The small size of urban rivers and the excessive construction of sluices will undoubtedly affect the material transport movements in rivers and then the river environment [5]. The factors affecting the water quality of the regulated rivers are more complex and are susceptible to the influence of water diversion and storage by sluices and dams [6]. They can largely result in the degradation of water quality in rivers [7]. The water quality of rivers in cities directly or indirectly affects human health, and thus the health of regulated rivers and the attainment of water quality standards are essential to the high-quality development of the urbanization process [8].

According to the 2020 statistics of the World Commission on Dams (WCD), the number of dams worldwide has reached 59,071, of which the number in China reached 23,841, accounting for about 40% of the world [9]. Urban rivers play a significant role in water extraction, navigation, and stormwater drainage, and also in urban domestic sewage as well as part of the industrial wastewater discharge. Furthermore, the impact of sluices and dams on river environments and ecology is increasing for watersheds with high population density, relatively concentrated production and life, and more serious water pollution [10]. Especially in some areas with abundant stormwater, various flood control sluices and dams have been built in urban rivers to prevent urban waterlogging. These sluices and dams inevitably affect the hydrological status of the rivers, thereby affecting the diffusion and distribution of pollutants in urban rivers. Studies have shown that the accumulation of nitrogen and phosphorus pollutants without treatment will bring great risks to the water environment [11].

Currently, there are many studies focusing on the impact of sluice and dam construction on the water quality of rivers. Dou et al. [12] analyzed the effect of sluice operation on water quality in the Shaying River and developed a hydrodynamic model incorporating sluice operation and a water quality transport and transformation model that incorporated the release of endogenous loads and identified that the influent concentration, size, and the number of sluices were the main factors affecting water quality. Wang et al. [13] reported the effect of sluice operation around Poyang Lake on water quality and found that sluice operation slowed down the water flow rate and increased the risk of water eutrophication. Young et al. [14] found that the opening of the Arase Dam resulted in a significant decrease in the concentrations of As, Zn, Pb, and the total sulfur in the mudflat sediments of the Kuma River and the aquatic environment improved. Tang et al. [15] demonstrated that the construction and operation of a large number of sluices in the Yangtze River Basin changed the natural transport rhythm of the runoff, suspended solids and nutrients, and reduced flow velocities, resulting in the decline of water exchange, the narrowing of the connectivity between rivers and lakes, and the accumulation of nutrients and SS, which led to water eutrophication. Soukhaphon et al. [16] concluded that the sluices in the Mekong River Basin affected fish migration, river hydrology, and sediment transport and consequently had a negative impact on regional food economic security. Obviously, the impact of sluices and dams on the river water environment is multifaceted. However, the environmental effects of large dams or sluices are universally known, but those of these small sluices or dams (≤15 m or ≤3 × 10^6^ m^3^) have rarely been considered [17]. In view of the proliferation of flood-prevention dams in the world’s river systems, the challenge appears as to their cumulative impacts on water environments. An endeavor to evaluate these flood-prevention facilities’ cumulative environmental impacts suggested that a large number of small dams or sluices may have an immeasurable impact on energy generation than that of large ones [18]. Thus, there is an urgent requirement to understand the multiple environmental impacts of small flood-prevention development and to understand how these dams or sluices might be better developed and managed.

This study focuses on urban regulated rivers in Wuxi, and the water quality data of rivers within the city are compared and analyzed to explore the differences in the changes of major pollutant concentrations upstream and downstream of urban rivers when the sluices are closed, and then to explore the impact of small sluices construction and operation on urban rivers water quality. The harmonious balance between the urban water environment and ecology is a critical basis for sustainable urban social and economic development; however, the interaction between them is extremely complex [19]. The target of China’s water resources management has been changing from “water quantity management” to “water quality management” [20]. The results of this study can provide a basis for the management of water resources in urban rivers, ecological regulation, and the construction of small sluices and dams and promote the coordinated development of socio-economic and urban rivers.

## 2. Materials and Methods

### 2.1. Study Area

Wuxi is one of the cities with numerous rivers in China, and it is located on the north shore of Taihu Lake in the Yangtze River Delta [21]. There are more than 3100 rivers in Wuxi, with a total length of 2480 km. The total length of the rivers in the city is approximately 150 km, with the volume of the water body being 8 million m^3^ during the flat water period. Wuxi is relatively rich in surface water and is well recharged by external water sources. The storage capacity of the city is 63.49 million m^3^, and the annual recharge is 64.53 million m^3^ [22]. Wuxi has now built more than 1200 large and small sluices because of rapid industrialization and urbanization. In 2020, Jiangsu Province invested about 5.9 billion dollars focusing on the implementation of flood control projects in the Taihu Lake Basin, which has greatly improved the flood control capacity of Taihu Lake [23]. The construction and operation of these new sluices and dams, as well as the regulation of existing ones, will have a direct impact on the water quality of Wuxi’s rivers and Taihu Lake.

**Figure 1:**
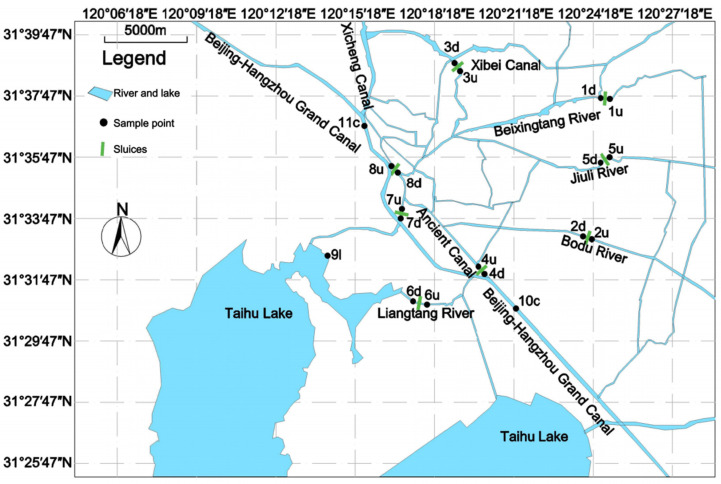
Research area and sample points.

Currently, about 50% of rivers in Wuxi fail to meet the requirements of Class III “Water Environmental Quality Standards of China” (WEQSC) (GB3838-2002). Among them, the ones in Wuxi’s downtown were the most seriously polluted, and the main pollution indicators are total nitrogen (TN) and total phosphorus (TP) [24]. The pollution sources in Wuxi are mainly municipal solid waste, industrial pollution, and agricultural irrigation and fertilization. In this study, we selected 8 major rivers with a total length of 159.34 km (Figure 1). Among them, Bodugang River, Xibei Canal, Jiuli River, and Liangtang River, and their water quality are required to meet the Class III of WEQSC (TN ≤ 1.0 mg·L^−1^; TP ≤ 0.2 mg·L^−1^). Meanwhile, the water quality of the Xicheng Canal, Beijing–Hangzhou Grand Canal, Beixingtang River, and Ancient Canal is required to meet Class IV (TN ≤ 1.5 mg·L^−1^, TP ≤ 0.3 mg·L^−1^) (GB3838-2002).

A new “Flood Control Plan of Wuxi” was issued at the end of 2001, which would protect a 136 km^2^ region. In May 2003, the construction of flood control facilities began and was completed at the end of 2008 [22]. This project contained eight flood control stations: Yandaigang flood control station, Beixingtang flood control station, Jiuli River flood control station, Bodugang flood control station, Limin Bridge flood control station, Xianli Bridge flood control station, Liangtang flood control station, and the Jiangjian flood control station. These stations are applied not only to prevent floods in Wuxi but are also used for ship navigation [25]. The floodgates are controlled by the relevant government departments according to the annual precipitation and total water quantity of Wuxi City.

### 2.2. Sampling and Experiment

In this study, the main rivers in Wuxi city were selected, and the sampling points were concentrated in eight flood control stations. Point 1 was at Beixingtang River, point 2 was at Bodugang River, point 3 was at the Dongting Maritime Section of Xibei Canal, point 4 was at the Ancient Canal, point 5 was at Jiuli River, point 6 was at Liangtang River, point 7 was at the Water Conservancy Bureau section of the Huancheng River, and point 8 was at the Canal Park section of the Huancheng River. Points 1–8 include two points upstream and downstream, respectively. For example, point 1 includes 1u and 1d, and 1u means point 1 upstream, and 1d means point 1 downstream (Figure 1). The water samples were collected and analyzed for the upstream and downstream areas of the eight flood control stations (Figure 1). In addition, sampling site No. 9 is located in Taihu Lake, and No. 10 and No. 11 are located in the Beijing–Hangzhou Grand Canal. These three water samples were used to compare and analyze the water quality differences between the urban rivers and Taihu Lake and the Beijing–Hangzhou Canal when the sluices were closed.

This study was conducted from 2018 to 2019, including the flat-water season, wet season, and dry season. A suitable temperature was required to reduce the interference from external factors, such as rainstorms, on water quality. Meanwhile, when collecting the water samples, the water samples were taken 0.5–1.0 m below the water surface and far away from the river shore to reduce the impact of edge effects due to the shallow rivers. The sluices were all closed when the samples were collected.

Before sampling, the river water was taken to clean and moisten the water extractor. Then, the polyethylene storage bottle was washed more than 3 times by the water in the water extractor, and the water sample was immediately taken full and put into the cryogenic storage box for preservation. In order to reduce errors and to simulate the real situation as much as possible, the number of water samples was six at each point, and all water samples were measured three times. After sampling, all of the water samples were placed in the laboratory refrigerator at 4 °C, and all of the water quality data were measured within 24 h.

TN and TP are significant indicators for evaluating the water quality of the Taihu Lake Basin to meet WEQSC (GB3838-2002) [26]. Therefore, TN and TP were selected as the water quality indicators in this study. According to the national standards of HJ636-2012 and HJ671-2013, the detection of TN concentration adopts the “Alkaline potassium persulfate ablation UV spectrophotometric method” [27], and TP adopts the “Ammonium molybdate spectrophotometric method” [28].

### 2.3. Methods

#### 2.3.1. Relative Difference

This study introduces the concept of “relative gap” to compare and analyze the difference between the upstream and downstream pollutant concentrations [29]. It is defined as:

(1) $$\;DR=\frac{Cu-Cd}{Cu}\times 100\%$$

where *DR* is the relative difference between the pollutant concentrations upstream and those downstream, *Cu* is the pollutant concentration upstream, and *Cd* is the pollutant concentration downstream.

If the calculated result is negative, it means that the pollutant concentration downstream of the river is higher than that upstream, while if it is positive, it means that the pollutant concentration downstream is lower than that upstream.

#### 2.3.2. Gaussian Fitting

In order to more deeply quantify the difference between pollutant concentrations upstream and downstream when the sluices are closed, the frequency distribution of the relative difference of pollutant concentrations was fitted using the Gauss fitting method in the normal distribution model in OriginLab software. The formula is as follows:

(2) $$y=y_{0}+\frac{A}{w\sqrt{\pi /2}}e^{\frac{-2{\left(x-x_{c}\right)}^{2}}{w^{2}}}$$

where *y*_0_ is the baseline, *x*_0_ is the mean, *w* is the discrete degree parameter, and *A* is the shape parameter.

## 3. Results and Discussion

### 3.1. Variation of Water Quality Indicators in Different Seasons

**Figure 2:**
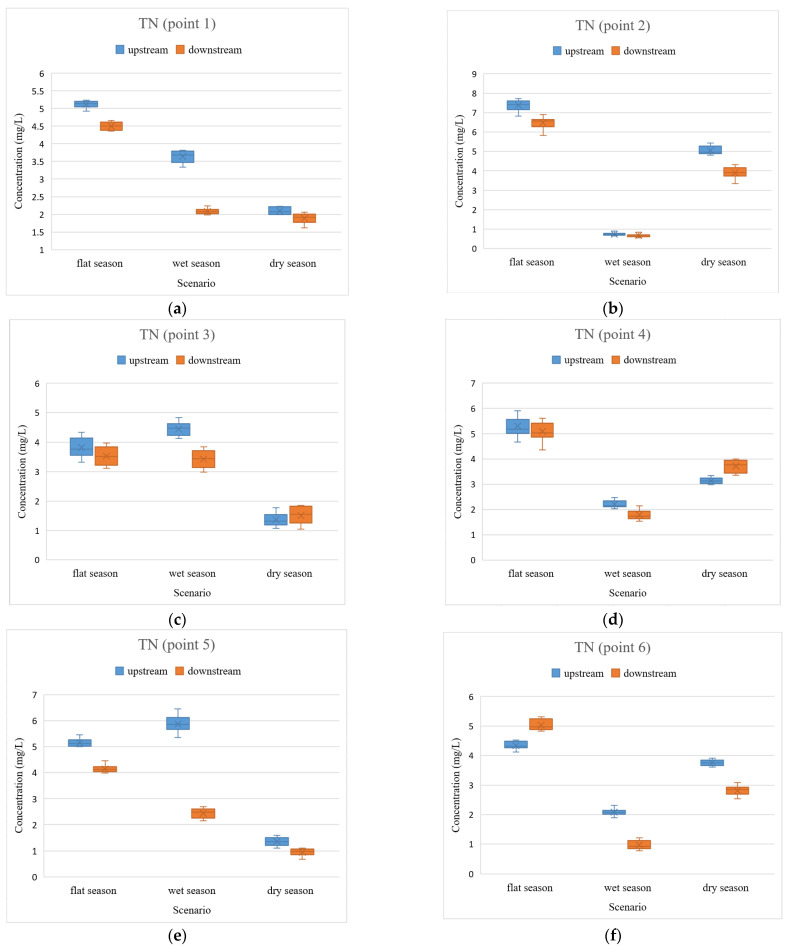

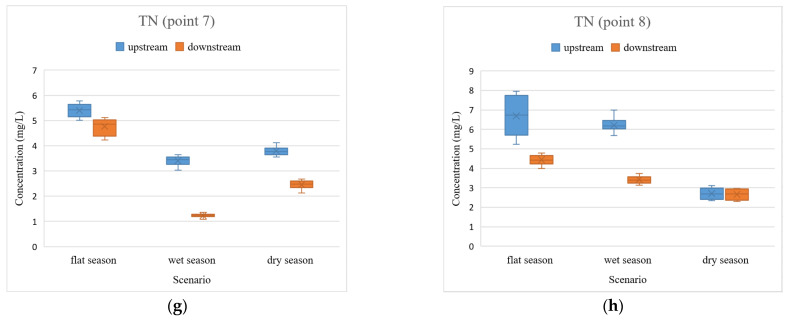
Comparison of TN upstream and downstream of the rivers in the closed state of the sluices. (**a**) TN concentrations at point 1; (**b**) TN concentrations at point 2; (**c**) TN concentrations at point 3; (**d**) TN concentrations at point 4; (**e**) TN concentrations at point 5; (**f**) TN concentrations at point 6; (**g**) TN concentrations at point 7; (**h**) TN concentrations at point 8.

**Figure 3:**
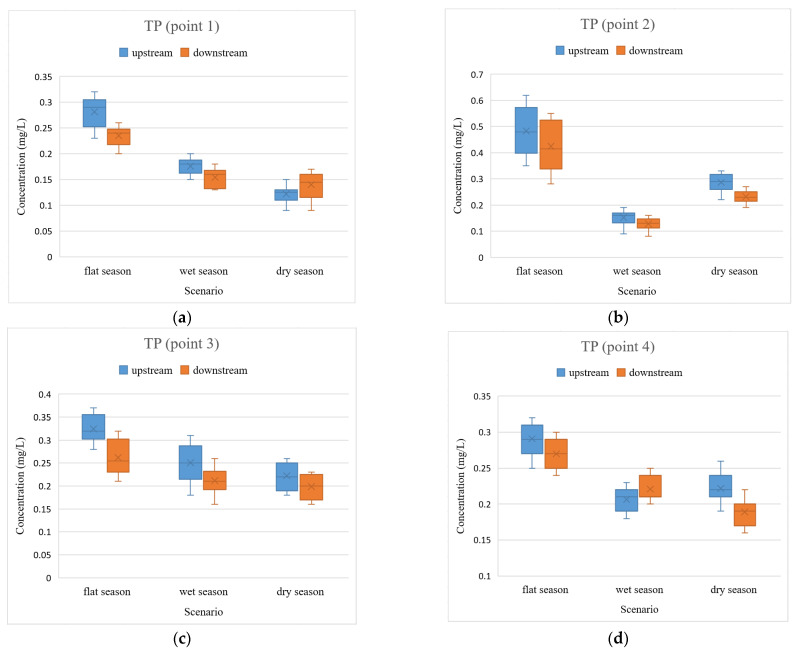

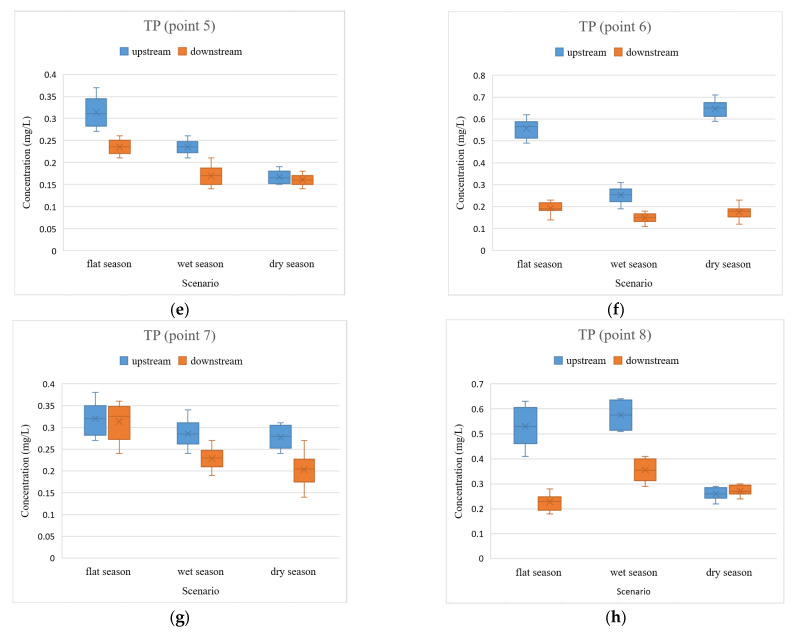
Comparison of TP upstream and downstream of the rivers in the closed state of the sluices. (**a**) TP concentrations at point 1; (**b**) TP concentrations at point 2; (**c**) TP concentrations at point 3; (**d**) TP concentrations at point 4; (**e**) TP concentrations at point 5; (**f**) TP concentrations at point 6; (**g**) TP concentrations at point 7; (**h**) TP concentrations at point 8.

Urban rivers often take into account the role of discharging domestic sewage, most of which are nutrient pollutants. Nutrient pollutants mainly include nutrients represented by nitrogen and phosphorus, which are not considered pollutants in themselves. However, when the level of nutrients contained in sewage is relatively high, it will contribute to the proliferation of algae in the water and eutrophication of the water body, which leads to a series of hazards [30]. Furthermore, some studies have indicated that the main pollutants in inlet and outlet rivers around the whole of Taihu Lake are dominated by nitrogen pollutants, followed by organic pollution such as phosphorus [31]. Combined with the cyanobacterial water pollution events that have occurred in Taihu Lake [32], TN and TP are selected as water quality indicators in this study. The comparison results of TN and TP are shown in Figure 2 and Figure 3.

The concentrations of TP and TN were relatively low during the wet and dry seasons compared to those in the flat-water season due to the fact that the wet and dry seasons are the summer and autumn seasons in Wuxi, with more rainfall. On the one hand, the high precipitation leads to the high storage capacity of rivers and the constant turnover of the water body. These are conducive to pollutant concentration reduction. On the other hand, for flood control purposes, the sluices are opened to release flood water when there is excessive rainfall. Furthermore, the river flows faster, and some pollutants from the river will flow into larger water bodies such as the Beijing–Hangzhou Grand Canal, which contributes to reducing pollutant concentrations.

In addition, there are some cases of excessive pollutant concentrations during the wet and dry seasons; for example, the TN concentrations upstream of the rivers in the wet seasons (Figure 2c,e,h) and the TP concentrations upstream of the rivers during the wet and dry seasons (Figure 3f,h), respectively. This is due to the fact that summer and autumn are not only the peak season of precipitation in Wuxi but also the peak period for industrial production and domestic sewage discharge. The combination of effluent discharge, sluice closure, and high temperatures during periods brings about high concentrations of these pollutants, especially during the time when it does not rain. This result is similar to previous studies [33].

### 3.2. Variation of Water Quality Indicators in the Upstream and Downstream

To further analyze the variation in the pollutant concentrations upstream and downstream of the rivers, the relative differences in the pollutant concentrations were calculated by Equation (1). The relative differences upstream and downstream of the rivers for TN and TP pollutants are shown in Table 1 and Table 2.

The 24 groups of TN concentrations are compared in Table 1. Two groups of TN concentrations in the upper sites were smaller than those in the lower sites, and the relative difference between the upstream and downstream was not obvious. While another 22 groups of TN concentrations upstream were higher than those downstream, among which 13 groups, relative differences ranged from 0 to 30% between upstream and downstream, the relative differences of the five groups ranged from 30 to 100%, and four groups relative differences exceeded 100% between the upstream and downstream.

The results indicated that the water quality in the upper reaches of the river was worse than that in the lower reaches when the sluice status was closed, with the maximum relative difference between the upper and lower reaches of the TN concentration being greater than 100%. Meanwhile, the TN concentrations were relatively high in all rivers. There were a few rivers where the differences in TN concentrations between the upper and lower reaches were not significant, but the comparison of the differences in TN concentrations between the upper and lower reaches in most rivers was very obvious.

The TP concentration in the rivers of Wuxi was relatively lower compared to TN (Figure 2 and Table 2). Among the 24 sets of data, there were eight sets of relative differences within 10%, which is not a significant comparison. Furthermore, among the remaining 16 sets of data, the TN concentrations in three sets were in the upper reaches, less than those in the lower reaches, and the other 13 sets in the upper reaches were greater than that in the lower reaches. Meanwhile, of these 16 sets, there were seven sets with relative differences between upstream and downstream from 10% to 30%, six sets were between 30% and 100%, and three sets where the difference exceeded 100%. The concentration of TP was not high in general, and some of the data were not obvious enough for a clear comparison. However, for the 16 groups of data, it can still be concluded that the water quality in the upper reaches of the rivers is worse than that in the lower reaches.

Generally, the concentrations of TP were relatively low, and the degree of variation in TP was not as great as that of TN. Furthermore, the levels of TP and TN also differed significantly at different times of the year at the same site in the same river, reflecting seasonal variability. In addition to this, an important preliminary conclusion was drawn: the pollutant concentrations in the upper reaches of the Wuxi rivers were higher than those in the lower reaches when the sluices were closed. The result is highly consistent with the previous investigations [34,35].

**Figure 4:**
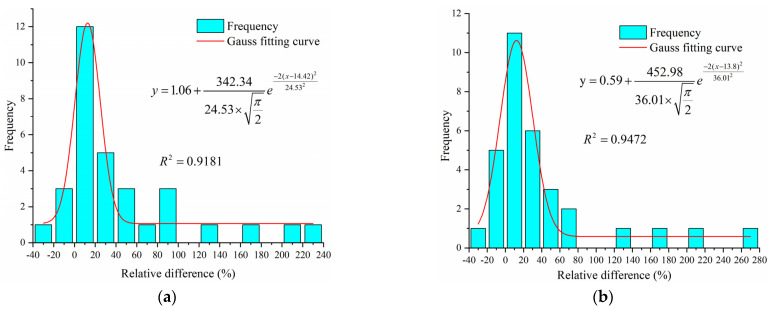
Relative difference frequency distribution and Gauss fitting curve (**a**) TN (**b**) TP.

In order to further quantitatively analyze the pollutant concentrations in the upper and lower sites of rivers, the interval length of 20% was firstly selected for the frequency distribution map for frequency distribution statistics, and then the frequency distribution was fitted by applying Equation (2). The specific frequency distribution plots, as well as the fitted curves, are shown in Figure 4.

The value of the Gauss fitting curve parameter xc for the relative disparity frequency of TN was 14.42 (Figure 4). That is, under the normal distribution model, the average value of the relative differences between the TN concentrations in the upper and lower reaches when the sluices were closed was 14.42%. Similarly, the mean value of the relative differences in TP concentration was 13.80%. This indicates that the TN and TP concentrations in the upper reaches of urban rivers in Wuxi were 14.42% and 13.80% higher than those in the lower reaches under the closed state of the sluices, respectively. This is consistent with our preliminary conclusions.

### 3.3. Variation of Water Quality Indicators in Urban Rivers

When the sluices are closed, the water quality upstream of urban rivers may be worse than that downstream. The reason for this phenomenon is inextricably linked to the water quality conditions of the urban rivers themselves.

First of all, the pollution sources of urban rivers have special characteristics compared to the general large rivers such as the Yangtze River and the Yellow River, has a great distinction. Some studies have concluded that the main pollutants in the Yangtze River come from urban domestic sewage and agricultural pollution [36]. Furthermore, the cross-sections below the Three Gorges Dam are mainly located in the main urban living area, which causes some pollutant indicators in the lower reaches of the sluices and dam to be significantly higher than those in the upper reaches [37]. For urban rivers, in Wuxi, the city is located in the middle and upper reaches of rivers, where domestic sewage and industrial sources are the most important sources of nutrients and pollution [38]. These effluents flow into the rivers from the middle and upper sites, and these pollutants accumulate in the upper reaches of the sluices when they are closed. The presence of numerous sluices leads to the accumulation of large amounts of industrial wastewater, domestic sewage, and solid waste sediment in the upper reaches of the sluices. Although domestic, agricultural, and industrial wastewater is also discharged into the middle and lower reaches of urban rivers, the lower reaches usually connect to larger water bodies, such as Taihu Lake and the Beijing–Hangzhou Grand Canal. Consequently, the water quality upstream of urban rivers is often worse than that downstream.

**Figure 5:**
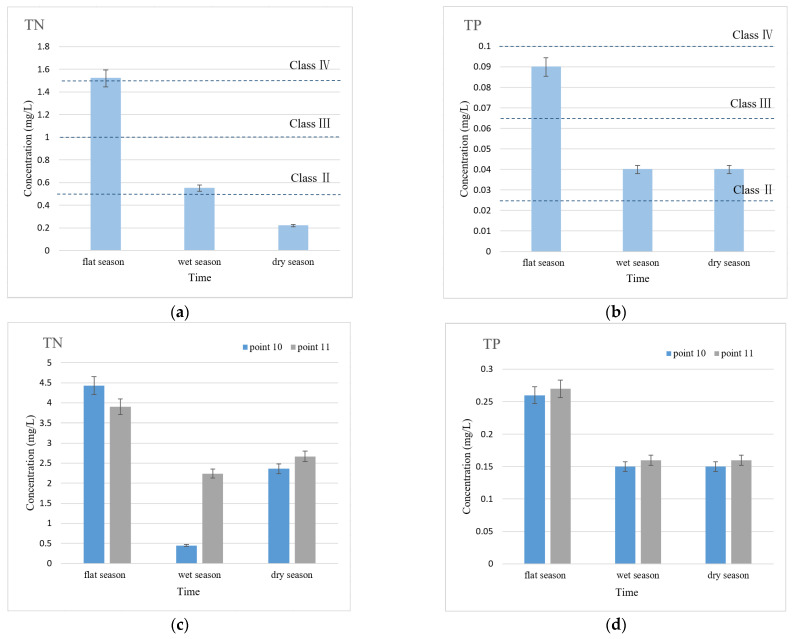
TN and TP concentration in Taihu Lake and Beijing–Hangzhou Grand Canal. (**a**,**b**) Point 9 at Taihu Lake; (**c**,**d**) Point 10 and point 11 at Beijing–Hangzhou Grand Canal.

Secondly, the lower reaches of urban rivers connect to larger water bodies, which own large volumes and lightly polluted water, as well as relatively clear water quality compared to that of urban rivers, and the concentrations of TN and TP are also relatively low. The concentrations of TN and TP in Taihu Lake and the Beijing–Hangzhou Grand Canal are shown in Figure 5.

It can be seen from Figure 5 that although the concentrations of the TN and TP in Taihu Lake and the Beijing–Hangzhou Grand Canal have partially exceeded the standards of Class III or Class IV WEQSC (GB3838-2002), they were still much lower than the average levels of TN and TP concentrations in urban rivers, as shown in Figure 2 and Figure 3. In addition, the water storage volume of Taihu Lake and the Beijing–Hangzhou Grand Canal are more than other rivers in the city, and their dilution effect on pollutants is more obvious. Therefore, when the downstream of the river is connected with these water bodies with relatively clear water quality and huge water volume, the pollutants can be effectively diffused and decomposed, and the water quality downstream of the sluices is better than upstream.

## 4. Conclusions

The effect of the construction of sluices on the water quality of urban rivers in Wuxi was investigated, and the difference between TN and TP in the upper and lower reaches of the urban rivers after the construction of the sluices was detected. In this paper, the water quality in the urban rivers showed obvious seasonality and was usually better in seasons that have more rainfall, such as summer and autumn. However, irregular sluice regulation often causes some water quality pollutant concentrations to rise abnormally. Additionally, in the state of sluice closure, the water quality of the urban rivers upstream was worse than that downstream; the concentrations of TN and TP downstream were, on average, 14.42% and 13.80% lower than that upstream, respectively. The concentrations of pollutants showed different degrees of variation with time and space, and there were discrepancies between the data and conclusions at individual monitoring sites. For example, the pollutant concentrations in the Ancient Canal and Huancheng River were higher downstream than upstream as well as the relative difference between the upstream and downstream pollutant concentrations in the Liangtang rivers was extremely obvious. In the future, additional sample sites and numerous data will be required for in-depth exploration.

## Figures and Tables

**Table 1:** Relative difference in TN concentration.

Points	Relative Difference (%)		
	Flat	Wet	Dry
1	12.0	83.3	8.7
2	17.7	0	29.7
3	7.1	37.5	−10.9
4	6.3	27.2	−20.4
5	26.4	219.7	52.6
6	222.8	125.3	28.3
7	14.3	175.2	57.3
8	71.8	90.6	0.7

**Table 2:** Relative difference in TP concentration.

Points	Relative Difference (%)		
	Flat	Wet	Dry
1	20.8	12.5	−7.1
2	9.4	0	26.1
3	10.7	19.0	10.0
4	7.4	−4.6	20.0
5	37.5	41.2	0
6	205.3	73.3	260.1
7	2.9	34.8	52.6
8	160.1	60.0	−3.3

## Data Availability

Not applicable.
